# An in-progress, open-label, multi-centre study (SAILOR) evaluating whether a steroid-free immunosuppressive protocol, based on ATG induction and a low tacrolimus dose, reduces the incidence of new onset diabetes after transplantation

**DOI:** 10.1186/2047-1440-3-12

**Published:** 2014-06-13

**Authors:** Jana Ekberg, Henrik Ekberg, Bente Jespersen, Ragnar Källen, Karin Skov, Michael Olausson, Lars Mjörnstedt, Per Lindnér

**Affiliations:** 1 Transplant Institute, Institute of Clinical Sciences, Sahlgrenska Academy at University of Gothenburg, Sahlgrenska University Hospital, Gothenburg, Sweden; 2 Department of Transplantation, Skane University Hospital, Malmoe, Sweden; 3 Department of Nephrology, Aarhus University Hospital, Skejby, Denmark

**Keywords:** diabetes, immunosuppression, renal transplantation

## Abstract

**Background:**

Corticosteroids and calcineurin inhibitors (CNIs) are included in renal transplantation immunosuppressive protocols around the world. Well-known side effects are associated with the use of these drugs, including new onset of diabetes after transplantation (NODAT). Long-term patient survival rates are lower among patients with NODAT. The optimal immunosuppressive protocol would therefore include not using corticosteroids and minimization of CNI use.

**Methods/Design:**

This is a prospective, multi-centre, controlled, randomized, parallel group, open-label study involving kidney transplant patients. The study compares a steroid-free immunosuppressive protocol (study arm A), which is based on low-dose tacrolimus and mycophenolate mofetil (MMF) maintenance therapy together with antithymocyte globulin (ATG) induction, with the conventional immunosuppressive protocol (study arm B), being based on low-dose tacrolimus, MMF and steroids together with interleukin-2 receptor (IL2-R) induction. The study is designed to include most normal-risk patients. It will exclude patients seen as at a high risk of rejection. The primary objective of the study is to assess the cumulative incidence of NODAT in the two study arms 12 months after transplantation using the American Diabetes Association type 2 diabetes diagnostic criteria. The composite measure of freedom from acute rejection, graft survival and patient survival will be evaluated. Renal function and chronic changes in the transplanted kidney will be assessed.

**Discussion:**

If this study confirms conceptual expectations, namely decreased incidence of NODAT, the steroid-free study protocol could be used with all patients. The regimen could be especially beneficial for patients at a high risk of diabetes mellitus.

**Trial registration:**

Trial registration: EudraCT 2012-000451-13.

## Background

Corticosteroids and calcineurin inhibitors (CNIs), such as tacrolimus and cyclosporine, are the main ingredients in immunosuppressive renal transplantation protocols throughout the world. These drugs have recognized side effects, such as nephrotoxicity, an increased incidence of diabetes, cardiovascular morbidity and malignancy. New immunosuppressive drugs, such as mycophenolate mofetil (MMF) and interleukin-2 receptor (IL2-R) antibodies now make it possible to reduce, withdraw or avoid corticosteroids or CNIs in many protocols [1]. Steroid-free protocols can now be used safely, providing that a standard trough level of tacrolimus is achieved and combined with MMF and IL2-R antibodies [2]. Doses of CNIs can also be reduced safely, as recently shown in the large Symphony trial, in which the best graft function was achieved in the study arm that used low-dose tacrolimus [3]. However, as in most other CNI-minimization protocols, corticosteroids were not discontinued.

New onset of diabetes after transplantation (NODAT) is associated with increased graft failure and mortality due to cardiovascular events [4]. Long-term patient survival is lower for patients with diabetes mellitus or NODAT when compared with non-diabetic patients [5]. The incidence of NODAT is variously described as reaching from 14% ten weeks after renal transplantation [6] to 37% one year after transplantation [7], and NODAT has been a constant challenge in tacrolimus-based immunosuppression since the drug was first introduced. The incidence of NODAT has decreased as tacrolimus doses have been reduced; but the incidence still seems to be higher than for cyclosporine-based regimens. The DIRECT study showed that the risk of NODAT or impaired fasting glucose is common in CNI-based regimens but is significantly lower with cyclosporine than with tacrolimus in the first 6 months post-transplant [8]. This is one reason cited by transplantation physicians as to why they use cyclosporine rather than tacrolimus with post-transplant patients perceived to be at risk of diabetes. As tacrolimus is a staple drug in modern immunosuppression [3], efforts to reduce NODAT rates have focused on steroid withdrawal. As corticosteroids induce insulin resistance, minimization is also likely to reduce NODAT.

The CARMEN study group reported a significant reduction of NODAT (0.4%) in a steroid-free arm when compared with a 5.4% rate for NODAT in a control arm [2]. The steroid-free arm used daclizumab, tacrolimus and MMF, while the control arm used tacrolimus, MMF and steroids, without IL2-R antibody induction. In contrast, other studies have proposed that basiliximab, also an IL2-R antibody, impaired glucose homeostasis and was associated with an increased risk of NODAT [9,10].

Antithymocyte globulin (ATG) has long been used as a potent induction agent in organ transplantation. It has been used in steroid-free [11,12] and CNI-minimization protocols [13]. To better reduce side effects and toxicity, the optimal immunosuppressive protocol would include both avoidance of corticosteroids and minimization of CNIs. No such protocols have appeared in the literature or been otherwise described. A future protocol, designed to avoid rejections due to inadequate immunosuppression will need to replace corticosteroids or CNIs by increasing or optimizing other immunosuppressive strategies. We believe that the use of ATG induction, instead of IL2-R antibodies, along with the use of therapeutic drug monitoring of MMF, would fit into such a protocol.

The proposed study aims at addressing both the diabetogenic side effect of CNIs and corticosteroids as well as the cardiovascular side effects of corticosteroids in an avoidance protocol.

## Methods/Design

**Figure 1:**
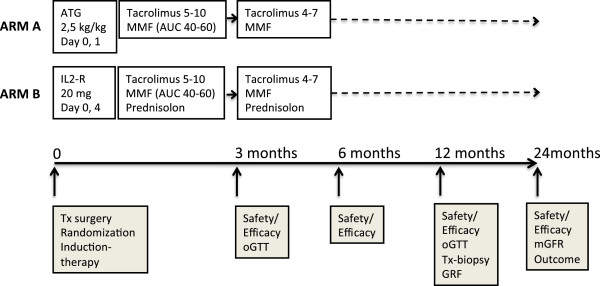
**Treatment and follow-up schedule in the SAILOR-study.** ATG, antithymocyte globulin; AUC, area under the plasma concentration time curve; IL2-R, interleukin-2 receptor; mGFR, measured glomerular filtration rate; MMF, mycophenolate mofetil; oGTT, oral glucose tolerance test; Tx, transplant.

This is a prospective, multi-centre, controlled, randomized, parallel group, open-label study of participants who are enrolled in kidney transplant programmes. The study is comparing a steroid-free immunosuppressive protocol based on low-dose tacrolimus and MMF maintenance therapy together with ATG induction (study arm A) with a conventional immunosuppressive protocol based on low-dose tacrolimus and MMF and steroids together with IL2-R induction (study arm B). The study design is illustrated in Figure 1.

Prior to transplantation, a representative group of 222 *de-novo* kidney transplant recipients will be randomized to either of the two study groups (111 in each group) after signing an informed consent agreement. Assignment of subjects to treatment groups will be stratified by donor status (living versus deceased) and by centre.

Enrolment will continue until the required sample size has been randomized. An enrolment time of 36 months is expected.

The study includes persons above 18 years of age who are receiving a first or second single kidney transplant from a deceased or living donor and who are considered able to benefit from a standard immunosuppressive protocol. The study participants are able to give written informed consent and each individual will need to agree to participate in the study for 24 months.

Patients will be excluded from participation if they: (1) already have a diagnosis of diabetes mellitus or have a plasma glucose level of >11.1 mmol/l at admission; (2) are receiving steroids at the time of transplantation; or (3) are likely to need steroids after transplantation. Recipients of multiorgan transplants, those previously transplanted with any other organ than a kidney, or prospective study participants with a complement dependent cytotoxicity panel reactive assay >25% in their most recent tests are also excluded from the study. If the responsible investigator considers the study candidate, for any other reason, to be at a high risk of rejection (which requires enhanced immunosuppression) then the candidate will be excluded. Patients receiving a renal transplant from a human leucocyte antigen (HLA) identical sibling and patients who are recipients of ABO-incompatible kidney transplants will also be excluded from the study.

### Objectives and endpoints

The primary objective of the study is to assess the cumulative incidence of NODAT in the two study arms 12 months after transplantation. The NODAT diagnosis being used is adapted from the American Diabetes Association criteria for type-2 diabetes [14]. The diagnosis endpoint is measured as the cumulative incidence of:

•Fasting plasma glucose between 2 and 7.0 mmol/l, 30 consecutive days or more apart;

•2-hour plasma glucose ≥11.1 mmol/l in the oral glucose tolerance test, 30 days or more apart;

•Use of oral hypoglycaemic agents for 30 consecutive days or more;

•Insulin treatment for 30 consecutive days or more.

The oral glucose tolerance test will be administered after 3 and 12 months. If either of the two oral glucose tolerance tests is pathological, the reading will be confirmed by being repeated after 30 ± 7 days.

Secondary objective measures include other NODAT time points and any use of antidiabetic medication. The composite measure of freedom from acute rejection, graft survival and patient survival will be evaluated after 12 and 24 months. The incidence of antibody-mediated rejection using the Banff 2009 classification [15] and of donor-specific HLA antibodies will be analyzed after 3 and 12 months. Renal function will be evaluated by measured glomerular filtration rate mGFR and by Iohexol or Cr-EDTA clearance at 12 and 24 months. The incidence of chronic changes will be analyzed by protocol biopsies at 12 months, using the Banff 2009 classification system.

The incidence of hypertension, number and type of antihypertensive drugs and of lipid-lowering drugs will be evaluated at different time points.

### Sample size calculation

In two recent phase 3 studies with tacrolimus, steroids and MMF [7], the proposed definition of NODAT gave an incidence rate of 36%. It is reasonable to estimate that a steroid avoidance regimen can reduce the incidence of NODAT to half of that rate. A changed induction regimen (Thymoglobulin rather than Simulect) is expected to reduce the number of rejections. As described earlier, the frequency of rejections has a positive correlation with the incidence of NODAT.

We predict that the percentage of subjects who reach the endpoint of NODAT after 12 months will be 18% in the study group arm and 36% in the control group arm.

Based on this assumption, 222 subjects are required for randomization to the two treatment groups in a 1:1 ratio (steroid-free: steroids) to achieve 80% power for the superiority comparison (Fisher’s exact test) on the intention-to-treat population of the primary endpoint between the two treatment groups, with a two-sided type I error of 5% and allowing for a 5% drop-out rate.

### Randomization and treatment

The study participants will be randomized before transplantation to one of the two following treatment arms using a 1:1 relation from an internet-based system.

Study arm A: the steroid-free low-tacrolimus study arm will receive Thymoglobulin® induction (2.5 mg/kg, pre-or peri-operatively on day 0 and 2.5 mg/kg on day 1) and maintenance therapy of Advagraf® with an initial preoperative dose of 0.1 mg/kg followed by 0.2 mg/(kg.day) orally in one dose (concentration: 5 to 10 ng/ml; after 3 months, 4 to 7 ng/ml); and MMF 1gx2 started preoperatively, controlled by a single AUC (area under the plasma concentration time curve) measurement, on day 10 ± 5 days, with a target AUC of between 40 and 60 (mg.h)/l). To diminish ATG side effects, 250 mg methylprednisolone will be given before ATG infusion on day 0 and 50 mg will be given before ATG infusion on day 1.

Study arm B: the standard low- tacrolimus study arm protocol consists of Simulect® induction 20 mg intravenously (day 0 and day 4) and maintenance immunosuppression with an initial preoperative dose of 0.1 mg/kg followed by Advagraf® 0.2 mg/(kg.day) orally in one dose (concentration: 5 to 10 ng/ml) and after 3 months (4 to 7 ng/ml). MMF 1gx2 is started preoperatively (controlled by a single AUC measurement, on day 10 ± 5 days, with a target AUC between 40 and 60 (mg.h)/l) and steroids are added according to hospital practice, but not less than 5 mg prednisolone daily after 6 months.

### Trial organization

Called the SAILOR-study, this is an investigator-initiated trial. Preliminary investigator meetings were organized by Sahlgrenska University Hospital, the sponsor of the study. Two Swedish transplant centres (Gothenburg, Malmö) and one Danish centre (Aarhus) are participating in the study.

Study drugs will be purchased by the participating hospitals. The investigation medicinal products will be exempt from the EU Clinical Trials Directive as they will be used within the terms of their marketing authorization. All treatments will be on an open-label basis.

Before recruitment commenced, the sponsor visited all sites to train relevant staff in the study procedures. The sponsor will closely monitor recruitment rates and the completeness of follow-up data. Sites will be monitored during recruitment and follow-up through a combination of on-site visits and central statistical monitoring.

A data monitoring committee (DMC) will be appointed. An independent group, outside the sponsor and steering group, will have this responsibility. The DMC will consist of one or two physicians and one statistician; none will have any other involvement with the study. For efficacy, the DMC should use O’Brian-Fleming group sequential boundaries. The DMC should also look for safety and conditional power when giving advice regarding continuation of the study. The DMC should start to look at the data after 40% of the subjects have completed the study.

The work of the DMC will be defined in a DMC charter. The sponsor and DMC members, before their first look at the data, should sign off this document when 40% of the subjects have completed the study.

The Regional Ethical Review Board in Sweden and Denmark have approved the study. To protect participants, the study was designed to comply with the World Medical Association’s current Declaration of Helsinki on ethical principles of human experimentation.

The most relevant safety parameters assessed within this study are incidence of diabetes mellitus, acute rejection, renal function, loss of graft, incidence of cardiovascular complications and events or incidence of malignancy. Data on adverse events outside these topics will not be collected.

Adverse events are recorded in the study’s case report form. Serious adverse events are recorded in the case report form, on a serious adverse events worksheet and in the participant’s separate medical record.

The Transplant Institute, Gothenburg will be responsible for all statistical programming and analysis, as well as statistical quality control and validation of programming and statistical analysis. The responsible biostatistician will coordinate the statistical analysis.

A detailed description of all the statistical analyses of all efficacy and safety variables together with an overview of tables and figures will be given in a separate statistical analysis plan. The plan will be finalized before the database of the study is locked. Any deviations from the plan will be justified in the clinical study report.

## Discussion

This controlled study was designed to evaluate whether a steroid-free, low-dose CNI protocol based on tacrolimus, induction with a high-bolus dose of ATG and therapeutic drug monitored MMF, may be used without reduction in efficacy or safety and with side effects minimized. The protocol is to be compared with a low-dose CNI (tacrolimus) protocol with steroids and IL2-R inhibitor for induction.

Calcineurin inhibitors lower insulin secretion while steroids contribute to insulin resistance. The β cell toxic effect when tacrolimus is used is, however, dose-dependent and reversible [16]. If the tacrolimus concentration is kept low, the risk for diabetes can be minimized [17]. The difference between tacrolimus and cyclosporine in lowering insulin secretion has been shown to be present only very early after transplantation, but not later on [18]. The Symphony study sub-analysis describes no significant difference in fasting glucose between low-tacrolimus protocol and both cyclosporine protocols [19]. In the FREEDOM trial, which used a cyclosporine-based regimen, the *de-novo* use of antihyperglycemic medication was reduced from 14.7% to 4.5% when steroids were avoided [20]. While diabetogenic effects of steroids and CNIs are well documented, the role of IL2-R inhibitors in impairment of glucose homeostasis have been discussed, but the results are controversial [9,10].

The incidence of NODAT is dependent on how it is defined. Using the American Diabetes Association definition of NODAT, we are expecting rates above 30% in the steroid arm. We have not included HbA1c in diagnostic endpoints for NODAT, as it could be affected in early transplant period by anemia, uremia and erythropoietin usage [21].

With early steroid withdrawal, one retrospective study describes less rejection with ATG induction when compared with IL2-R antibody blocker use [12]. Usually, the drug is administered for 7 to 10 days after transplantation. There is, however, clinical [22] as well as experimental [23,24] evidence that suggests that a single high-bolus dose of ATG given just before transplantation has a similar effect; a dose might even have tolerogenic properties. In a large single-centre cohort, post-transplant diabetes was significantly reduced when steroid-free immunosuppression was given and the frequency in the steroid-free group was only 1% after 1 year [25].

Dosing of MMF may be optimized by increasing the initial dose [26], and by the use of therapeutic drug monitoring by estimated AUC measurements [27]. Optimized usage of MMF can also reduce the risk of rejection in a steroid avoidance regimen.

In the experimental arm, participants will receive a more powerful induction treatment (Thymoglobulin); however, they will not receive steroids. We do not expect the experimental arm to be less effective in preventing rejection and we expect that the steroid-free regimen will reduce the rate of diabetes.

Study patients will undergo a biopsy after 1 year. Biopsies are frequently performed, but not on all patients outside a study. The risk for bleeding after biopsy is low but sometimes a short period of haematuria is observed. Evidence is increasing that surveillance biopsies not only give information on acute rejection and chronicle changes but also add other important information for the care of the individual being treated [28,29]. An evaluation of implantation biopsies will help to distinguish donor-related changes from recipient-related processes.

Study participants will also undergo an oral glucose tolerance test, which is not associated with any side effects, other than the discomfort of drinking a strongly sweet fluid.

If this study confirms the conceptual expectations, namely a decreased incidence of NODAT, the steroid-free study protocol could be implemented among all study participants. The regimen could be especially beneficial among those with a high risk of diabetes mellitus or significant cardiovascular complications and among those with obesity, psychiatric conditions or skeletal demineralization. Implementation, of course, has to be balanced with a theoretically higher risk of rejection; the maintenance therapy is limited to two drugs and could be considered weaker than the conventional triple regime. A stronger induction therapy, with potential tolerance-induced effect, is used to compensate for the lessened maintenance immunosuppression. Signs of early acute rejection will not be the only function monitored; chronic changes, low-grade humoral rejection in protocol biopsies at 1 year and any development of donor-specific HLA antibodies will also be closely observed, to detect any sign of long-term under-immunosuppression. The current study has a two-year follow-up period and, therefore, cannot give answers concerning long-term patient and graft survival. We will consider extending the follow-up period to 5 years if the outcome is as expected.

## Abbreviations

ATG: antithymocyte globulin; AUC: area under the plasma concentration time curve; CNI: calcineurin inhibitor; DMC: data monitoring committee; HLA: human leucocyte antigen; IL2-R: interleukin-2 receptor; MMF: mycophenolate mofetil; NODAT: new onset of diabetes after transplantation.

## Competing interests

None of the authors has any competing interests.

## Authors’ contributions

PL, LM, MO and JE designed the study. PL, JE, MO and BJ drafted the manuscript. HE, BJ, RK, KS and MO contributed to the design of the study during several meetings. All authors read and approved the final manuscript.
